# Supplementary material for: Beat Detection Recruits the Visual Cortex in Early Blind Subjects
Source: Life (Basel). 2021 Mar 31;11(4):296. doi: 10.3390/life11040296 (PMC8066101; doi:10.3390/life11040296)
Supplement: Supplementary file 1 [file life-11-00296-s001.zip › life-1141981 supplementary.docx]

Article

Beat Detection Recruits the Visual Cortex in Early Blind Subjects

Rodrigo Araneda ^1^, Sandra Silva Moura ^1^, Laurence Dricot ^2^ and Anne G. De Volder ^1,^*

Supplementary Materials


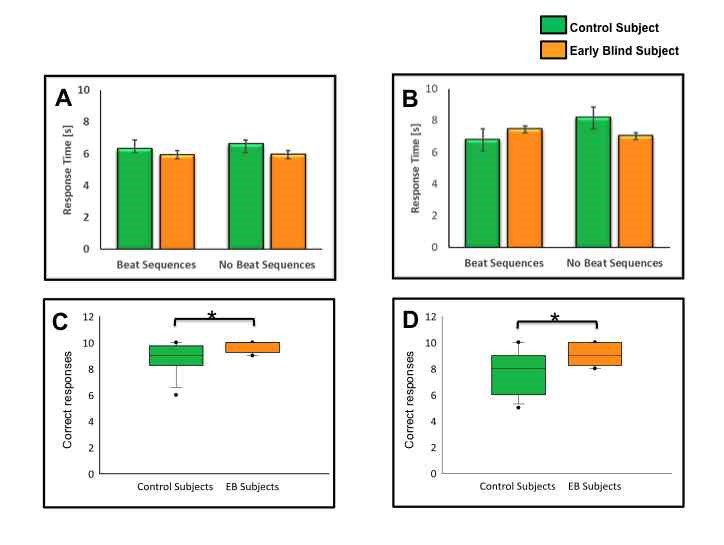


**Figure 1.** Graphical display of behavioral results.(**A**) Response times in auditory conditions. The mean response times for “beat/no beat” detection in auditory “beat” and “no beat” sequences were 6.32 ± 1.79 s. and 6.60 ± 2.10 s. in SC group and 5.93 ± 2.93 s. and 5.96 ± 2.97 s. in EB group. There were no significant difference between groups. (**B**) Response times in vibrotactile conditions. The mean response times for “beat/no beat” detection in vibrotactile “beat” and “no beat” sequences were 6.79 ± 2.46 s. and 8.17 ± 3.05 s. in SC group and 7.44 ± 4.04 s. and 7.03 ± 4.14 s. in EB group. The performance (reflected by shorter response times) was slightly better in the auditory modality in both groups, without significant difference between groups. (**C**) Response accuracy (median number of correct responses and confidence intervals) in auditory conditions. The median scores for “beat/no beat” detection (averaged from “beat” and “no beat” sequences) were 10.000 (25–75%: 9.250–10.000) for early blind participants and 9.000 (25–75%: 8.250–9.750) for controls. There were significant differences between early blind participants and their controls (p < 0.05, Mann-Whitney test). (**D**) Response accuracy (median number of correct responses and confidence intervals) in vibrotactile conditions. The median scores for “beat/no beat” detection (averaged from “beat” and “no beat” sequences) were 9.000 (25–75%: 8.250–10.000) for early blind participants and 8.000 (25–75%: 6.000–9.000) for controls. There were significant differences between early blind participants and their controls (*p < 0.05, Mann-Whitney test).

AudioS2_Beat_sequence.wav: Auditory version of a sequence used in the experimental condition “Beat”, where group units of a same inter-onset interval (IOI ‘1′, here 250 ms) and its integer multiples (IOI ‘x2′, ‘x3′ and ‘x4′) were temporally grouped in order to form units that were equal to the inter-beat-interval, each unit being delimited by a silent gap demarcating the intervals.

AudioS3_No_beat_sequence.wav: Auditory version of a sequence used in the experimental condition “no beat”, where one third of each IOI of the “beat” sequence was shortened by 30%, one-third remained the same and one-third was lengthened by 30%, these IOI’s being further randomly mixed to compose a sequence that was irregular and unpredictable.
